# Neoplasia-Associated Pericarditis—Predictor of Cancer Progression?

**DOI:** 10.3390/diagnostics11010058

**Published:** 2021-01-02

**Authors:** Anca Boldan, Alina Gabriela Negru, Maria Boldan, Laura Mazilu, Anca Tudor, Dorel Popovici, Sorin Săftescu, Constantin Tudor Luca, Șerban Mircea Negru

**Affiliations:** 1 Oncomed Oncology Outpatient Clinic, 300239 Timișoara, Romania; boldananca@gmail.com; 2 Department of Cardiology, University of Medicine and Pharmacy, 300041 Timișoara, Romania; 3 University of Medicine and Pharmacy Timișoara Student, 300041 Timișoara, Romania; office@oncohelp.ro; 4 Department of Oncology, Faculty of Medicine, Ovidius University of Constanta, 900470 Constanța, Romania; lauragrigorov@gmail.com; 5 Discipline of Computer Science and Medical Biostatistics, University of Medicine and Pharmacy, 300041 Timișoara, Romania; anca.ancutza@gmail.com; 6 Department of Oncology, University of Medicine and Pharmacy “Victor Babeș”, 300041 Timișoara, Romania; dorelpopovici@gmail.com (D.P.); sorin251@yahoo.com (S.S.); snegru@yahoo.com (Ș.M.N.)

**Keywords:** pericarditis, cancer, cancer progression, neoplasia, cardio-oncology, pericarditis marker of cancer progression/recidive

## Abstract

Pericarditis may signal the presence of cancer, even in the absence of other clinical or paraclinical signs. Corollary, the following question arises: Could the discovery of a newly developed pericarditis be used in patients with known neoplasia as a marker of cancer progression? In an attempt to find an answer to this question, this two-centre study included 341 consecutive patients with a confirmed diagnosis of cancer and evidence of pericardial effusion at echocardiography and/or CT/MRI scan. The patients’ data were collected retrospectively if they further fulfilled the following inclusion criteria: available medical data from confirmation of pericarditis until evidence of cancer progression or until at least 12 months without progression. The average age of the patients was 62.16 years (22–86 years), and the study comprised 44.28% males and 55.71% females. All types of the most common neoplasms were represented. The results showed that 85.33% of patients had cancer progression temporally linked to pericarditis. Of these, 41.64% had cancer progression within 18 months after the diagnosis of pericarditis with a median time to progression of 5.03 months, ranging from 0 to 17 months; 43.69% had progression within a maximum of 2 months before the diagnosis of pericarditis. Only 14.66% had no cancer progression during the observation period. We concluded that pericarditis could be a sensitive marker of cancer evolution that could be widely used as a follow-up investigation for cancer patients as a marker of progression or recidive.

## 1. Introduction

The pericardial sac contains an average volume of 10–50 mL of pericardial fluid considered to be an ultrafiltrate of plasma. The pericardium drains via lymphatic vessels to the mediastinal and tracheobronchial lymph nodes. The presence of pericardial effusion is not rare in cancer patients, and the link of the pericardium to the pleural lymphatics provides the basis for cancer-related pericarditis, more frequently in lung and other thoracic neoplasia [1].

Primary malignant tumours of the heart leading to pericarditis are rare: mesothelioma, fibrosarcoma, angiosarcoma, and teratoma [1,2]. Secondary metastatic tumours are a more common cause of pericardial disease (above all, lung, breast cancer, and lymphoma) [3].

The mechanisms by which cancer leads to the development of pericarditis are multifactorial:

– Direct infiltration by cancer cells from proximate structures;
– Pericardial haemorrhage;
– Spread of cancer cells to pericardium through the bloodstream;
– Cancer treatments: most frequently radiation therapy and a variety of antineoplastic drugs (pericarditis frequently associated with toxic cardiomyopathy: doxorubicin, daunorubicin, cytosine-arabinoside, cyclophosphamide, 5-fluorouracil and lately, immunotherapy) [4]. The localisation of the tumour, the direction of radiotherapy beam and dose >30 Gy are responsible for an increase in the pericarditis incidence in patients with a variety of cancers (left and right breast, lung, oesophagal) [5]. Radiotherapy-associated acute pericarditis is uncommon and generally associated with mediastinal tumours. Most cases resolve spontaneously [6,7].
– Decreased immunity from cancer itself or cancer therapies increasing the risk of opportunistic viral or bacterial infections;
– Paraneoplastic syndromes: an assembly of modifications triggered in response to the presence of a neoplasm, which are defined as clinical syndromes involving nonmetastatic systemic effects that accompany malignant disease [8]. These syndromes are collections of disorders that result apparently from two distinct pathogenic mechanisms: substances produced by the neoplasm and antibodies directed towards the tumour, causing a cross-reaction with other tissues [9]. They are typically associated with lung, breast, lymphatic, ovarian, testicular cancer, or teratoma.

The term “pericarditis” suggests inflammation as the primary pathophysiological mechanism, although this is not always the case. For simplification, this paper will use the term “pericarditis” for any pericardial effusion, irrespective of the underlying pathophysiology. Standard classifications of pericarditis were used to define the size (mild <10 mm, moderate 10–20 mm, and large >20 mm), distribution (circumferential or loculated), and the haemodynamic effect (with or without tamponade) [3,10].

The concept of this paper started with our observation during the work in the cardiology practice that patients with neoplasia who also present with various degrees of pericarditis have more frequent follow-up appointments. The higher follow-up frequency was linked to changes in the chemotherapeutic line due to cancer progression (cardiological re-evaluation requested at the initiation of a new chemotherapeutic drug).

We also observed that most cancer patients already having pulmonary/thoracic metastasis have various degrees of pericarditis. These range from minor quantities, which are hardly visible during diastole, to significant liquid volumes leading to cardiac tamponade. However, most cases are pericarditis with small amounts of fluid, asymptomatic, and with no clinical consequences and no need for specific treatment.

Based on these observations, the following question arises: Could the occurrence of pericarditis in cancer patients be a marker heralding progression? Could pericarditis be a two way street for clinicians—that is, not only cancer triggering for the search of pericarditis but also pericarditis triggering a search for progression of cancer?

This issue could have important practical implications, such as the need for more frequent oncological follow-up (including CT/MRI) or more sensitive investigations (PET-CT) for the early detection of progression in such patients, sometimes despite the appearance of the stabilised disease.

Some extra support for this theory is the knowledge that among patients with pericardial effusion and no known cancer, malignancy is more prevalent, ranging between 12% and 23% of pericarditis cases. The link was observed in a large population-based cohort study which was based on several Danish national medical databases and included patients admitted to hospital with pericarditis over 20 years (1994–2013). The cancer follow-up started upon admission for pericarditis and continued for up to 20 years. The risk of receiving a cancer diagnosis after the hospital admission for pericarditis was compared with the cancer risk in a population with a similar age and gender. The risk was 12-fold higher than expected within the first three months. The fact that pericarditis may be the first clinical manifestation of concealed cancer, most frequently lung cancer, lymphoma, leukaemia, and other unspecified metastatic cancer, is one of the study’s most relevant results. Kidney, prostate, bladder, ovary, and colon cancers also were detected shortly after pericarditis diagnosis. The risk of a newly diagnosed cancer was highest within the first three months after the pericarditis diagnosis, and it was most notable among patients with wet pericarditis (pericardial effusion) [2]. However, it is reasonable to think that if pericarditis could indicate a concealed cancer, it could also indicate a hidden progression of known cancer.

## 2. Materials and Methods

Starting from the premises mentioned above, we intended to find out how many of our pericarditis patients develop cancer progression that can be timely linked to the first diagnosis of pericarditis.

Our study included patients addressed to the cardiology department of Timișoara Oncohelp Oncology Center (313 patients, 91.78%) and patients addressed to the Constanța Oncology Department of Clinical Emergency Hospital, Romania (28 patients, 8.21%).

Virtually all oncologic patients addressed to the Cardiology Department of Timișoara Oncohelp Oncology Hospital and all oncologic patients addressed to the Constanța Oncology Department of Clinical Emergency Hospital are investigated by echocardiography at baseline examination and periodically after. The frequency of echocardiograms depends on several variables: age, comorbidities, type of cancer, oncological therapy, and stage of cancer.

The medical files of all these patients with a confirmed diagnosis of cancer who were investigated at the cardiology practice starting July 2014 until March 2020 were examined. All patients who had evidence of pericardial effusion at the echocardiographic examination and/or CT/MRI scan were selected. For these patients, relevant data from their files were collected in an attempt to determine the nearest cancer progression episode related to the moment of diagnosis of pericarditis. Hematologic cancers have been excluded, although an essential number of pericarditis was observed in our practice in patients with such pathology.

The echocardiographic examination comprised 2D imaging of all standard views (parasternal long and short axis, apical 4, 5, 2 and 3 chamber view). The subcostal view was used in the rare cases of poor thoracic echographic window or skin lesions that impeded transthoracic examination. Subcostal view and/or unconventional views were also applied if necessary for better characterisation of the circumferential extension and maximum thickness of the pericardial fluid.

For moderate/large, circumferential, or loculated amounts of pericardial effusion, the diagnosis of pericarditis was evident, and the maximum thickness measured in diastole was reported.

In case of small amounts of fluid, particular attention was paid during the examination to differentiate between a clinically significant effusion and the normal pericardial fluid (echo-free space located at the posterior atrioventricular groove, usually only during systole) or the coronary sinus. Therefore, effusions that were visible exclusively at the level of the posterior atrioventricular groove in either parasternal or apical short-axis views and visualised either in systole and diastole or only in systole were considered physiological and were not reported as pericarditis. If the effusion extension was beyond the atrioventricular groove, along the posterior ventricular wall towards the apex (as highlighted from the parasternal long-axis view) or the lateral ventricular wall towards the apex (visible from the apical 4 or 5 chamber view), it was reported as “minimal”.

Of note, in case of significant amounts of pericardial fluid, pericardiocentesis was performed if clinical (tachycardia, dyspnea, hypotension, oedema, pulsus paradoxus) and echocardiographic (swinging heart, right atrium/ventricle collapse, significant respiratory variations of intracardiac and inferior vena cava flows) signs of tamponade were also present. The decision for intervention was taken based on clinical judgement integrating the present signs of tamponade and not merely based on the measurements of the pericardial fluid. As the procedure was performed in the cardiac surgery department of a different hospital, results from the pericardial fluid analysis are not available. Pericardiocentesis meant only for diagnostic purposes (cytology/biochemistry/neoplastic markers) is not locally available, so it was not performed in the absence of cardiac tamponade.

The patients’ data were collected retrospectively if they further qualified as follows: available medical data from confirmation of pericarditis until cancer progression or until at least 12 months without progression; patients who were lost to follow-up before these time points were excluded from our statistics. The maximum time of medical data for evaluation (if these data were available) was 24 months to confirm stabilisation of the disease.

The timely relationship between the diagnosis of pericarditis and the diagnosis of progression has been established. Three different situations were distinguished:

Progression after the moment of pericarditis diagnosis; if the progression appeared 0–18 month after pericarditis, it was considered a relevant time relationship between the two. All progression beyond 18 months was considered too late to be relevant.

Progression before pericarditis diagnosis; not all patients had a cardiologic evaluation at baseline (at the time of cancer confirmation). Therefore, these patients had no echocardiography result available previous to CT confirmation of progression. As the number of these patients is substantial and we observed that echocardiographic evidence of pericarditis was in most cases close in time to the moment of progression diagnosis, we considered this timely relation relevant. Individually, it was considered relevant if the investigation confirming progression was performed within a maximum of 1 month before the echocardiography that confirms pericarditis (for patients receiving specific cancer therapy) or within a maximum of 2 months before the echocardiography that confirms pericarditis (for patients receiving no cancer therapy). The rationale for this is that specific cancer therapy could alter the evolution of cancer in two months but much less probably in 1 month. The judgement was that no significant change in cancer status could have occurred in 2 months without treatment that could alter the results of the study significantly. In short, once progression was confirmed 0–2 months before the echocardiography, it can be assumed that there is a relation between progression and pericarditis.

Either progression occurred more than 2 months before the diagnosis of pericarditis (more than 1 month before for patients receiving cancer therapy), or more than 18 months after the diagnosis of pericarditis—in these cases, no relationship was considered to exist between pericarditis and progression.

Some specific data collected and processed during the study were compared to a control number of patients data retrieved for Romania on GLOBOCAN site (Global Cancer Observatory) [10].

Statistical analysis was performed with SPSS software, (version 17, SPSS Inc., Chicago, IL, USA). The data were electronically filed using Microsoft Excel (version 2013, MS Corp., Redmond, Washington, DC, USA). For numeric variables, descriptive statistics were performed, and the comparisons between these were made with the non-parametric Kruskal–Wallis test for more than 2 series with no Gaussian distribution, and the Mann–Whitney test was used for comparisons between two sets of values with no Gaussian distribution. For nominal variables, frequency tables were elaborated, and the associations between these were achieved by applying the chi2 (χ2) test. The results were considered significant for a value of *p* < 0.05.

## 3. Results

The total number of patients included in our study was 341.

The average age of the patients was 62.16 years (22–86 years). There were 44.28% male (151) and 55.71% female (190) patients.

**Figure 1:**
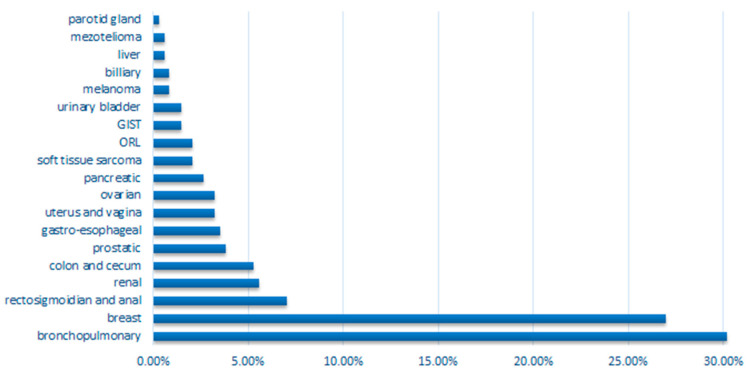
Graphical representation of the proportions of cancer types in our study population.

The proportions of bronchopulmonary, breast, renal, sarcoma and mesothelioma cancers are significantly increased in our study when compared to global cancer statistics (GLOBOCAN). The proportions of prostatic, oto-rhino-laryngological (ORL), urinary bladder, and liver cancers are significantly lower compared to the proportions described in the literature (*p*-values and significance are shown in Table 1 and Figure 1).

The quantity of pericardial fluid ranged from minor (difficultly visible during diastole) to 3 cm. Small and medium quantities were asymptomatic, whereas large quantities were associated with symptoms and even clinically manifest cardiac tamponade.

The following statistics were calculated from our database: (1) percentage of patients with progression of cancer immediately before (1 or 2 months) or within 18 months after the diagnosis of pericardial effusion; and (2) type of progression: specific location of progression and also the percentage of extrathoracic vs intrathoracic progression.

The results are presented below.

Percentage of patients with progression:

41.64% of patients (142 cases) had progression within 18 months after the diagnosis of pericarditis; the median time to progression was 5.03 months, ranging from 0 to 17 months;

43.69% of patients (149 cases) had progression before the diagnosis of pericarditis (as specified above);

85.33% of patients had cancer progression temporally linked to pericarditis;

14.66% of patients (50 cases) had no cancer progression during the observation period.

The age values are not significantly different between the three samples (Kruskal–Wallis Test, *p* = 0.177), but the P (mm) values are significantly different (Kruskal–Wallis Test, *p* = 0.002). The P (mm) values are significantly increased for the patients who had progression before the diagnosis of pericarditis compared with the patients who had no cancer progression (Mann–Whitney U Test, *p* = 0.003), also for the P (mm) values for the patients who had progression within 18 months after the diagnosis of pericarditis (Mann–Whitney U Test, *p* < 0.001). Instead, between the two types of cancer progression, the P (mm) values are insignificantly different (Mann–Whitney U Test, *p* = 0.588). The results are summarised in Table 2.

**Figure 2:**
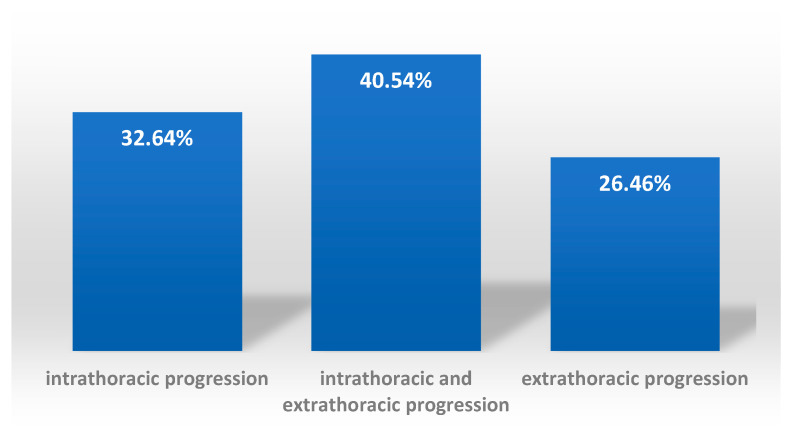
Distribution of progression type by localisation—intrathoracic, extrathoracic and both intrathoracic and extrathoracic, in patients with pericarditis.

Distribution of type of progression:

32.64% (95 cases) of patients had exclusively intrathoracic progression;

40.54% (118 cases) of patients had intrathoracic and extrathoracic progression;

26.46% (77 cases) of patients had exclusively extrathoracic progression; of these, 21.30% (62 cases) had abdominal progression, and only 5.15% (15 cases) had exclusively other sites of progression (Table 2), i.e., extrathoracic and extra-abdominal. The results are summarised in Figure 2.

Of note, some of the patients had more than one site of progression, with either multiple intrathoracic *or* extrathoracic sites, or combined intrathoracic *and* extrathoracic progression.

The specific sites of intrathoracic progression are presented in Table 3. Extrathoracic progression sites are listed in Table 4.

Distribution of progression in correlation with the quantity of pericardial fluid:

21.99% of the patients with progression had only minor pericarditis (64 patients);

78% of the patients with progression had ≥0.3 cm of pericardial fluid, as measured in diastole;

38% of the patients without progression had only minor pericarditis (19 patients);

62% of patients without progression had ≥0.3 cm of pericardial fluid, measured in diastole;

88.65% of the patients with ≥0.3 cm of pericardial fluid had progression.

Among patients with progression-associated pericarditis, only 6.52% (19 cases) received thoracic radiation therapy in the last 6 months before the diagnosis of pericarditis. For the patients without progression, the percentage of radiation therapy in the last 6 months before pericarditis was 16% (8 patients). Therefore, we concluded that radiation-induced pericarditis could have been present only in a minority of cases and could not have been a bias to the study. However, radiation therapy could explain the pericardial reaction in patients without progression; this is sustained by the higher percentage of associated radiation therapy of the thorax in these patients.

The Kruskal–Wallis test has been used to identify the significance of age values in four samples of patients: with intrathoracic progression, extrathoracic progression, intrathoracic and extrathoracic progression, and without progression. The Mann–Whitney U test has been used to establish the significance of the quantity of pericardial fluid measured in millimetres (mm) at echocardiography in patients without progression, with intrathoracic progression, with extrathoracic progression, or with both intrathoracic and extrathoracic progression (Table 5).

The age values are not significantly different between the four samples (Kruskal–Wallis Test, *p* = 0.075), but the P (mm) values are significantly different (Kruskal–Wallis Test, *p* < 0.001).

The P (mm) values in the non-progression case are significantly lower than the P (mm) values for the other cases (intrathoracic, extrathoracic, and both)—(Mann–Whitney U Test, *p* < 0.001).

The P (mm) values in the extrathoracic progression case are significantly lower than the P (mm) values for the intrathoracic and extrathoracic case (Mann–Whitney U Test, *p* = 0.021).

## 4. Discussion

Pericarditis represents as low as 0.2% of all cardiovascular admissions with many subclinical or missed diagnostically cases [12]. This is also the case of pericardial disease and cardiac function in cancer, which lack structured and constant diagnostic and follow-up approaches before, during, and after treatment [4,12].

Although several studies have shown the link between pericarditis and occult cancer or newly diagnosed cancer, so far, there are not enough data to show the importance of pericarditis in early detection of cancer progression or recidive.

This study is the first to explore the role of pericarditis as a marker for cancer progression.

The most relevant conclusion of our study is that pericardial effusion appearance in a cancer patient seems to play a predictive role for future progression before the appearance of any other clinical or paraclinical clue.

As much as 85.33% of patients were confirmed with cancer progression within 18 months or 1 or 2 months before the diagnosis of pericarditis.

A percentage of 73.19% of progressions included at least one intrathoracic site. If abdominal progression is added, the percentage rises to 94.5%. Only 5.15% of cases had exclusively extrathoracic-extra-abdominal progression.

Of note, the median time to progression among the patients with progression *after* diagnosis of pericarditis was 5 months in our study, which are results close to those found in newly diagnosed cancer in patients with pericarditis regarding the time of highest incidence (i.e., the first 3 months after the pericarditis diagnosis) in a Danish study [13].

The quantity of the pericardial fluid seems to be also of significance, as smaller quantities (minimal—i.e., not measurable or visible only in systole) were more frequent in the non-progression group (38% versus 21.99% in the progression group).

Women were slightly better represented among our patients with pericarditis (55.71% vs 44.28% men). The most plausible explanation could be the high prevalence of breast cancer, which is a thoracic sited cancer, among these patients.

It is very important that the percentage of bronchopulmonary and breast cancer in our population was significantly higher than the percentage of these types of neoplasms among cancer patients in Romania (i.e., 13.58% for bronchopulmonary and 11.53% for breast). What could be the physiopathological mechanism behind this finding? This observation could be a matter for further research, although some mechanisms could be figured out intuitively. For instance, could the anatomy of the lymphatic system, the “forgotten child of anatomy”, play a role in the phenomenon? Could the pericardium be considered a sort of “sentinel lymph node” for thoracic (and also nearby extrathoracic) progression?

The lymphatic vessels of the heart concretise in two plexuses: (a) one deep, situated immediately under the endocardium; and (b) one superficial, subjacent to the visceral pericardium. The deep plexus breaches into the superficial, the efferents of which form left and right collecting trunks. There are two or three left trunks, which rise in the anterior longitudinal sulcus, getting, in their route, vessels from both ventricles. On reaching the coronary sulcus, they connect with a large trunk from the diaphragmatic face of the heart and then combine to form a single vessel that ascends between the left atrium and the pulmonary artery and ends in one of the tracheobronchial glands. The afferents of the right trunk come from the diaphragmatic surface and the right border of the right ventricle and the right atrium. It rises in the posterior longitudinal sulcus and then runs forward in the coronary sulcus, and it passes up behind the pulmonary artery to end in one of the tracheobronchial glands [14,15].

It could be assumed that any blockage of lymph drainage at or above the tracheobronchial glands would impede the lymph drainage from the heart. As pericardium is a distensible space, fluid will accumulate inside it, and even small quantities can be immediately visible by different imagistic techniques. Thus, even small quantities of pericardial fluid could be the first objective sign of neoplastic involvement of the intrathoracic lymphatic system, even before the ganglia are enlarged enough to be considered affected. Thus, pericardial fluid could be a much more sensitive (and also probably more specific) reactant than the ganglia dimension, indicating neoplastic proliferation in the thorax. This feature can also apply not only to thoracic neoplastic invasion but also to anatomic areas that ultimately drain in the thoracic lymphatic vessels, such as the diaphragm, liver, and peritoneum (especially the upper peritoneum). This could also apply to lymphomas/leukaemias, where thoracic lymph nodes are involved, and where pericarditis is common (these patients were excluded from our study). The small percentage of patients that had exclusively extrathoracic and extra-abdominal progression seems to be a concordant sample of this theory.

Our observations are consistent with the lymphatic theory in the case of localised pericarditis. Anterior fluid accumulation is linked to progression in the right thorax, whereas posterior or lateral fluid accumulation is associated with progression in the left thorax. The distribution of drainage areas for lymphatic ganglia and vessels could explain this predilection [14].

The finding that among our pericarditis patients, lung and breast cancer (both being thoracic cancers) were more frequent and significantly better represented than in the general cancer patients in our country also sustains a physiopathological mechanism that most probably links to the anatomy of the thorax.

An important observation is that any tumour, if situated close enough to the pericardium, could trigger pericarditis, without progression being evident (example: mid-mediastinal bronchopulmonary tumours), carrying the meaning that the pericardial effusion is a reaction to any nearby neoplastic process, either the initial tumour (stable or progressive) or a metastasis. In the case of tumours close enough to the heart, a pericardial effusion could not necessarily mean progression of cancer, with a high probability that in those cases, the prognostic value of pericarditis could be reduced. This explanation could also sustain the situation of three cases with exclusively cerebral progression, all having bronchopulmonary cancer as the primary tumour.

Of note, there were a few cases with significant pericardial exudates but without neoplasia progression or intrathoracic tumour, where a crucial inflammatory process of the thoracic wall (such as postsurgical breast seroma or thoracic wall abscess) was observed. The theory of pericardium as a significantly enlarged “lymph node” could explain the pericarditis in these cases, too.

A significant drawback of the study is that CT/MRI evaluation was not always performed in a manner to diagnose early progression. The moment of imagistic evidence of progression is not necessarily the real moment of progression. Conversely, the moment of imagistic evidence of pericardial effusion is not necessarily the moment of actual appearance of the effusion, as pericarditis is asymptomatic in most cases, and echocardiography is usually performed at every 3–6 months, leaving the possibility of late diagnosis. Probably, a prospective study with a pre-established well-designed schedule of imagistic follow-up both for pericarditis and cancer progression would provide much more accurate data on the correlation between the moment of pericardial fluid appearance and the moment of cancer progression.

An important practical implication of the study could also be the following: Is it possible to monitor selected cancer patients only by echocardiography, which is much cheaper and much less invasive than radiation-based imagistic examinations? Close follow-up of the appearance of pericarditis by echocardiography could serve as a noninvasive screening tool that would trigger more detailed oncological work-up.

## 5. Conclusions

Pericarditis is frequently an accompanying feature of an anatomically nearby cancer, be it the primary tumour or a metastasis.

In the process of monitoring cancer patients, the appearance of a newly developed pericarditis could represent a red flag for progressive disease. Echocardiography is the most available, cheap, reproducible, and noninvasive investigation for detection of pericarditis, even for small amounts of pericardial fluid.

Pericarditis could be a sensitive marker for either the further evolution of already metastatic cancer, growth of the initial tumour, or the appearance of metastasis if initially absent—in short, any progression of the disease. Our results show that 85.33% of cancer patients with newly diagnosed pericarditis were confirmed with cancer progression, either within 18 months after or 1–2 months before the diagnosis of pericarditis. Intrathoracic progression seems to predominate (73.19% of progressions included at least one intrathoracic site). In addition, the quantity of the pericardial fluid appears to be essential for prognosis (88.65% of the patients with ≥0.3 cm of pericardial fluid had progression, while 21.99% of the patients with progression had only minor—<0.3 cm—pericarditis).

The cardiac function requires continuous follow-up, especially in cancer patients before, during, and after chemotherapy or radiation therapy. Echocardiography is a complete, available, and affordable investigation. In perspective, echocardiography can be used as a screening method for pericarditis, which seems to be not only a marker of occult cancer but also a recurrence marker of already known neoplasms, making it possible to detect recidive more accurately and to establish the appropriate and early therapy. The echocardiography is widely accessible, portable, and provides a complete noninvasive cardiac structural and functional hemodynamic assessment.

Further clinical research could establish the role of pericarditis detection by echocardiography, which is possibly an essential tool for monitoring cancer progression.

## Figures and Tables

**Table 1:** The distribution of cancer types in the study population compared with the actual known data extracted from the Global Cancer Observatory (GLOBOCAN) [11].

Cancer Type	Absolute Frequency (*n* = 341)	Relative Frequency (*n* = 341)	Relative Frequency (*n* = 83.461)	p^sig^ (Chi^2^ Test)
Bronchopulmonary	103	30.20%	13.6%	<0.001
Breast	92	26.97%	11.5%	<0.001
Rectosigmoidian and anal	24	7.03%	5.96%	0.473
Renal	19	5.57%	2.4%	<0.001
Colon and cecum	18	5.27%	7.3%	0.182
Prostatic	13	3.81%	7.2%	0.021
Gastro-esophageal	12	3.51%	5.08%	0.232
Uterus and vagina	11	3.22%	3.06%	0.989
Ovarian	11	3.22%	2.2%	0.273
Pancreatic	9	2.63%	3.7%	0.367
Soft tissue sarcoma	7	2.05%	0.07%	<0.001
ORL	7	2.05%	4.41%	0.047
GIST	5	1.46%	1.24%	0.904
Urinary bladder	5	1.46%	4.7%	0.007
Melanoma	3	0.87%	1.3%	0.645
Billiary	3	0.87%	0.75%	0.954
Liver	2	0.58%	4.1%	0.002
Mezotelioma	2	0.58%	0.08%	0.023
Parotid gland	1	0.29%	0.21%	0.787

**Table 2:** Descriptive statistics for age and progression measurements, for three samples.

Parameters	Sample (Progression)	*n*	Mean	Std. Deviation	Std. Error	95% Confidence Interval for Mean		Mean Rank
						Lower Bound	Upper Bound	
Age	Without	50	59.7	10.79	1.53	56.6	62.8	146.38
	Before	149	62.5	10.52	0.86	60.8	64.2	172.82
	After	142	62.5	10.09	0.85	60.8	64.2	175.44
P (mm)	Without	50	0.5	0.58	0.08	0.3	0.7	126.47
	Before	149	2.0	4.03	0.33	1.4	2.7	174.92
	After	142	2.0	3.73	0.31	1.4	2.6	181.44

**Table 3:** Distribution of intrathoracic progression.

Localisation of Progression	Number of Patients	Percentage
Pulmonary	131	45.01%
Thoracic lymph nodes	70	24.05%
Pleural	68	23.36%
Bone (thoracic)	41	14.08%
Thoracic wall (muscle, bone, soft tissue, breast)	18	6.18%
Mediastinal	9	3.09%
Myocardium	1	0.34%

**Table 4:** Distribution of extrathoracic progression.

Localisation of Progression	Number of Patients	Percentage
Liver	88	30.24%
Abdominopelvic lymph nodes	44	15.12%
Peritoneum, including ascites and carcinomatosis	39	13.40%
Bone (extrathoracic)	33	11.34%
Abdominal organs (gastric, renal, pancreatic)	21	7.21%
Neck and axillary lymph nodes	20	6.87%
Brain	14	4.81%
Extrathoracic soft tissue	8	2.74%
Biochemical	6	2.06%
Pelvis	6	2.06%
Bone marrow	1	0.34%

**Table 5:** Descriptive statistics for age and P measurements for each type of progression localisation.

Parameters	Type of Progression	*n*	Mean	Std. Deviation	Std. Error	95% Confidence Interval for Mean		Mean Rank
						Lower Bound	Upper Bound	
Age	Without	58	59.8	10.66	1.40	57.0	62.6	147.02
	Intrathoracic	129	61.6	10.77	0.95	59.8	63.5	165.34
	Extrathoracic	84	64.0	8.86	0.97	62.1	65.9	189.38
	Both	68	62.6	10.96	1.33	59.9	65.2	174.51
P (mm)	Without	58	0.5	0.68	0.09	0.4	0.7	128.03
	Intrathoracic	128	1.9	3.79	0.34	1.2	2.5	183.26
	Extrathoracic	85	1.8	4.07	0.44	1.0	2.7	158.95
	Both	69	2.6	3.98	0.48	1.6	3.6	196.76

## Data Availability

Data is contained within the article.
